# HDAC class I inhibitor, Mocetinostat, reverses cardiac fibrosis in heart failure and diminishes CD90+ cardiac myofibroblast activation

**DOI:** 10.1186/1755-1536-7-10

**Published:** 2014-07-02

**Authors:** Hikmet F Nural-Guvener, Luidmila Zakharova, James Nimlos, Snjezana Popovic, Diego Mastroeni, Mohamed A Gaballa

**Affiliations:** 1 Cardiovascular Research Laboratory, Banner Sun Health Research Institute, 10515 W. Santa Fe Drive, Sun City, AZ 85351, USA; 2 L. J Roberts Center for Alzheimer’s Research at Banner Sun Health Research Institute, Sun City, AZ, USA

**Keywords:** Congestive heart failure, Myocardial Infarction, Myofibroblast, Mocetinostat, Fibrosis, HDAC, Rat

## Abstract

**Background:**

Interstitial fibrosis and fibrotic scar formation contribute to cardiac remodeling and loss of cardiac function in myocardial infarction (MI) and heart failure. Recent studies showed that histone deacetylase (HDAC) inhibitors retard fibrosis formation in acute MI settings. However, it is unknown whether HDAC inhibition can reverse cardiac fibrosis in ischemic heart failure. In addition, specific HDAC isoforms involved in cardiac fibrosis and myofibroblast activation are not well defined. Thus, the purpose of this study is to determine the effects of selective class I HDAC inhibition on cardiac fibroblasts activation and cardiac fibrosis in a congestive heart failure (CHF) model secondary to MI.

**Methods:**

MI was created by left anterior descending (LAD) coronary artery occlusion. Class I HDACs were selectively inhibited via Mocetinostat in CD90+ fibroblasts isolated from atrial and ventricular heart tissue *in vitro. In vivo*, Class I HDACs were inhibited in 3 weeks post MI rats by injecting Mocetinostat for the duration of 3 weeks. Cardiac function and heart tissue were analyzed at 6 weeks post MI.

**Results:**

In sham hearts, HDAC1 and HDAC2 displayed differential expression patterns where HDAC1 mainly expressed in cardiac fibroblast and HDAC2 in cardiomyocytes. On the other hand, we showed that HDAC1 and 2 were upregulated in CHF hearts, and were found to co-localize with CD90+ cardiac fibroblasts. *In vivo* treatment of CHF animals with Mocetinostat improved left ventricle end diastolic pressure and dp/dt max and decreased the total collagen amount. *In vitro* treatment of CD90+ cells with Mocetinostat reversed myofibroblast phenotype as indicated by a decrease in α-Smooth muscle actin (α-SMA), Collagen III, and Matrix metalloproteinase-2 (MMP2). Furthermore, Mocetinostat increased E-cadherin, induced β-catenin localization to the membrane, and reduced Akt/GSK3β signaling in atrial cardiac fibroblasts. In addition, Mocetinostat treatment of atrial CD90+ cells upregulated cleaved-Caspase3 and activated the p53/p21 axis.

**Conclusions:**

Taken together, our results demonstrate upregulation of HDAC1 and 2 in CHF. In addition, HDAC inhibition reverses interstitial fibrosis in CHF. Possible anti-fibrotic actions of HDAC inhibition include reversal of myofibroblast activation and induction of cell cycle arrest/apoptosis.

## Background

Interstitial fibrosis and fibrotic scar formation contribute to cardiac remodeling and loss of cardiac function in myocardial infarction (MI) and heart failure. In response to myocardial injury, the number of fibroblasts increased by replication of resident cardiac fibroblasts, recruitment of bone marrow cells and transformation of endothelial/epicardial cells to fibroblasts. Cardiac fibroblasts are activated leading to myofibroblasts, which are contractile, invasive, and high producers of extracellular matrix (ECM) proteins [1-3]. While myofibroblasts serve an important role in wound healing and scar formation to repair areas of cardiomyocyte loss, further deposition of interstitial ECM causes myocardial stiffness and loss of ventricular function. Therefore, elucidating the mechanism(s) that regulates cardiac fibroblasts may provide a therapeutic strategy to reduce the amount of fibrosis in heart failure.

Epigenetic alterations, such as histone modifications, have been shown to be involved in tissue fibrosis in multiple organs including kidney, lung, and heart [4]. Histone acetylation (by histone acetyl-transferases) relaxes normally tight chromatin super-coiling, enhancing accessibility of transcriptional regulatory proteins to promoter regions. On the other hand, histone deacetylases remove acetyl groups from lysine residues of histones and other proteins, which remodel chromatin, resulting in inhibition of gene expression. To date, 18 mammalian HDACs were identified and categorized into four classes. Class I HDACs (HDAC1, 2, 3, and 8) are widely expressed and have pro-hypertrophic function in heart disease. Among class I HDACs, HDAC1 and HDAC2 have been shown to play redundant roles in cardiac growth and function [4,5]. Several studies had reported that HDAC1 and 2 have pro-fibrotic roles in renal injury disease models [6,7]. Recently, an idiopathic pulmonary fibrosis study linked HDACs to myofibroblast differentiation and extracellular matrix deposition [8]. Moreover, small molecule HDAC inhibitors, especially Class I and II inhibitors, were shown to effectively retard myocardial remodeling, decrease interstitial fibrosis, and improve cardiac function in pathological heart conditions [9-13]. In most of these studies, HDAC inhibitors were applied in acute MI settings rather than after development of interstitial fibrosis in CHF. Thus, here we aim to investigate whether HDAC inhibition could be effective in reversing fibrosis in chronic conditions like ischemic heart failure. Here we explore the anti-fibrotic mechanisms of Mocetinostat, a highly specific HDAC1, 2, and 3 inhibitor, in cardiac fibroblasts.

In the present study, we demonstrated that HDAC 1/2 were upregulated in CHF infarcted areas and this upregulation progressed to non-infarcted myocardium including the left atrium when CHF advanced to a de-compensated stage. Using a small molecule HDAC inhibitor, Mocetinostat, we showed that inhibition of class I HDACs reversed interstitial collagen deposition and improved heart function in CHF. Moreover, Mocetinostat promoted reversal of myofibroblasts activation *in vitro*. In parallel, Mocetinostat treatment activated a p53/p21 axis and Caspase-3. Thus, the present study suggests that, in advanced heart failure settings, class I HDAC inhibition can reverse fibrosis. Anti-fibrotic activity of Mocetinostat includes reversal of myofibroblast phenotype and regulation of cell proliferation/apoptosis in cardiac fibroblasts.

## Results

### HDAC1 and 2 levels are elevated in CHF

**Figure 1:**
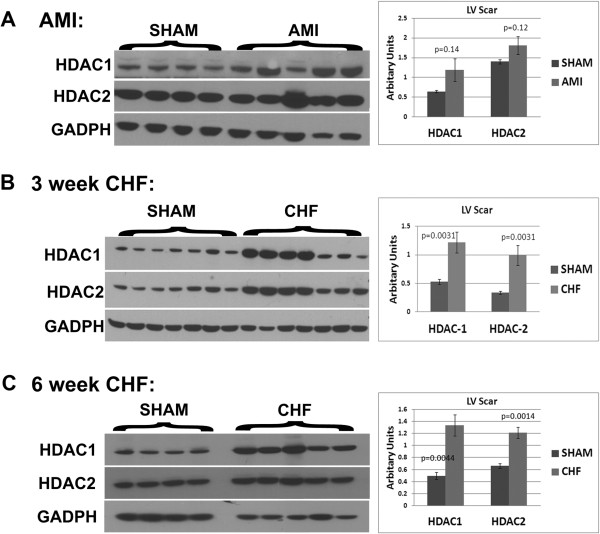
**HDAC1 and 2 levels are elevated in CHF.** Left ventricles from sham and infarcted myocardium at three time points: 3 days post MI (AMI), 3 weeks post MI (3w CHF), and 6 weeks post MI (6w CHF) were lysed and separated in 4% to 12% Bis-Tris SDS/PAGE gel and blotted with HDAC1 and HDAC2 antibodies. HDAC1 and 2 levels were normalized with GADPH levels and indicated as arbitrary units in bar graphs for each time points. Western blotting of the infarcted area showed no increase in HDAC1 and HDAC2 levels in AMI (n = 4 for sham, n = 5 for AMI) **(A)**. Significant increases in HDAC1 and HDAC2 levels were detected in the infarcted myocardium compared to sham in 3w CHF (n = 7 for both 3w sham and 3w CHF) **(B)** and 6 w CHF (n = 4 for 6w sham, n = 6 for 6w CHF) **(C)**. Error bars indicate S.E., *P* <0.05. CHF, congestive heart failure; HDAC, Histone Deacetylase.

**Figure 2:**
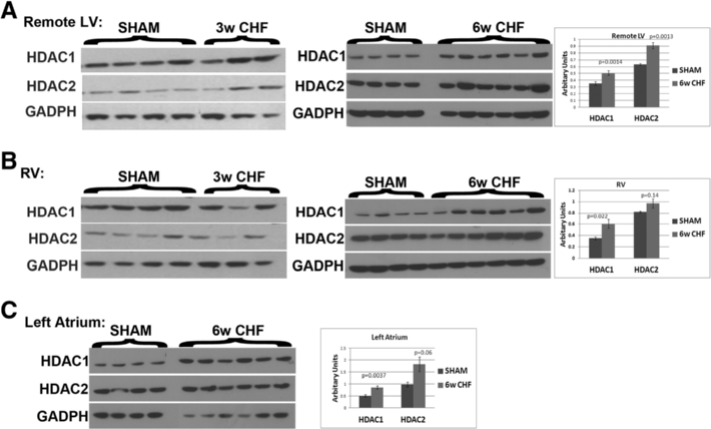
**HDAC1 and HDAC2 levels are elevated in non-infarcted myocardium in 6w CHF.** Non-infarcted myocardium (remote LV), RV and left atrium (LA) from sham, 3w CHF, and 6w CHF were lysed and separated in 4% to 12% Bis-Tris SDS/PAGE gel and blotted with HDAC1 and HDAC2 antibodies. GADPH levels were used as loading control. Western blotting of the remote LV showed significant increases in HDAC1 and HDAC2 levels compared to sham in 6w CHF **(A)**. Levels of HDAC1 were upregulated in 6w CHF RV **(B)**. Both HDAC1 and HDAC2 levels were elevated in left atrium in 6w CHF **(C)**. Error bars indicate S.E., *P* <0.05. CHF, congestive heart failure; HDAC, Histone Deacetylase; LV, left ventricle; RV, right ventricle. (n = 4 sham, n = 3 for 3w CHF) (n = 4 sham, n = 6 for 6w CHF).

First, we investigated the time course of HDAC 1 and 2 protein levels using western blot analysis at three time points following MI: at 3 days (Acute MI-AMI), 3 weeks (3w CHF) where the scar was formed and animals progressed into heart failure, and 6 weeks (6w CHF) where animals were in de-compensated heart failure [14] that is in junction with development of prominent fibrosis in the ventricles and atria. In AMI, both HDAC1 and HDAC2 showed a trend of increase in the infarcted myocardium (Figure 1A). At 3 weeks and 6 weeks after MI, HDAC1 and HDAC2 levels were upregulated in the infarcted LV (scar) (Figure 1B and C).In the non-infarcted myocardium, there was a trend of increase in HDAC1/2 levels in the LV of 3 w CHF (Figure 2A). The increase in HDAC1 and 2 levels in non-infarcted LV was significant for 6w CHF (Figure 2A). Moreover, only HDAC1 was upregulated in the right ventricle (RV) at 6w CHF (Figure 2B) whereas there were no changes in HDAC2 level at 3w CHF or 6w CHF (Figure 2B). HDAC1 levels were also unaltered in the RV at 3w CHF (Figure 2B).In 6w CHF hearts, left atria become enlarged and fibrotic. To investigate the association of HDAC1 and 2 with atrial fibrosis, we measured the levels of both proteins in left atrium (LA) in the 6w CHF group. Western blot analysis revealed elevated levels of both HDAC1 and 2 in the LA of 6w CHF compared to sham (Figure 2C). Thus, HDAC1/2 is upregulated in the infarcted area in CHF and progressed to non-infarcted myocardium and LA in de-compensated CHF.

### HDAC1 and 2 are co-localized with cardiac fibroblast in CHF

**Figure 3:**
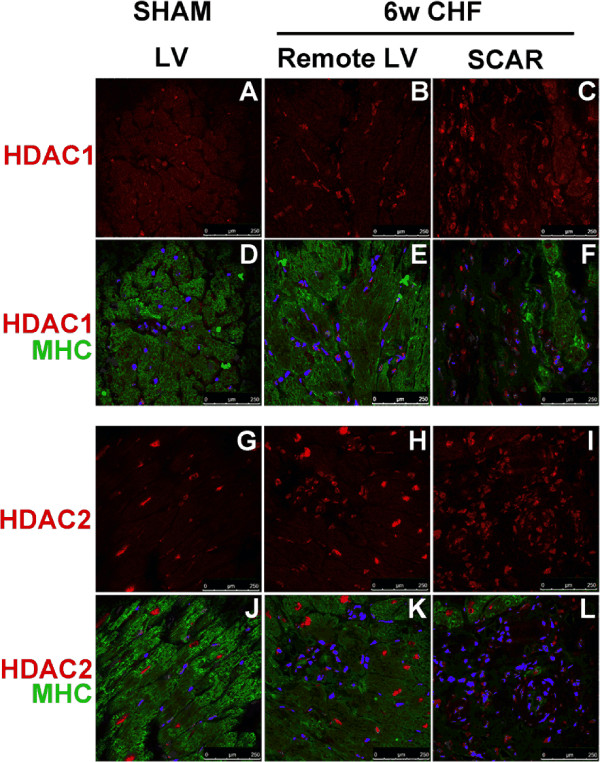
**Expression pattern of HDAC1 and HDAC2 in infarcted and non-infarcted LV.** Cross-sections of hearts from sham and 6w CHF rats were labeled with HDAC1 (red) **(A-F)**, HDAC2 (red) **(G-L)**, and α-MHC (green) antibodies. DAPI (Blue) is used to stain nuclei. HDAC1 was mainly expressed in interstitial cells, while HDAC2 was mainly expressed in cardiomyocytes in sham LV. In CHF, both HDAC1 and HDAC2 staining increased in interstitial cells in remote LV and infarcted area (Scar). Scale bars: 250 μm. α-MHC, Myosin heavy chain; CHF, congestive heart failure; HDAC, Histone Deacetylase; LV, left ventricle.

To investigate expression patterns of HDAC1 and 2, immunohistochemistry analysis was performed in axial and coronal sections of 6w CHF and sham hearts with corresponding antibodies against HDAC1 and HDAC2. In sham hearts, both HDAC1 and HDAC2 were uniformly expressed in the entire myocardium and atria. Co-staining of HDAC1 and HDAC2 with α-MHC showed that HDAC2 staining was more abundant in cardiomyocytes (Figure 3G, J) while HDAC1 staining was mainly in interstitial cells in between cardiomyocytes (Figure 3A, D). In CHF, both HDAC1 and HDAC2 staining were evident in interstitial cells in the non-infarcted LV (Figure 3B, E, H, K), while cardiomyocytes still had strong expression of HDAC2 (Figure 3K). In the infarcted myocardium, strong HDAC1 and HDAC2 staining was observed where α-MHC staining was diminished (Figure 3C, F, I, L).

**Figure 4:**
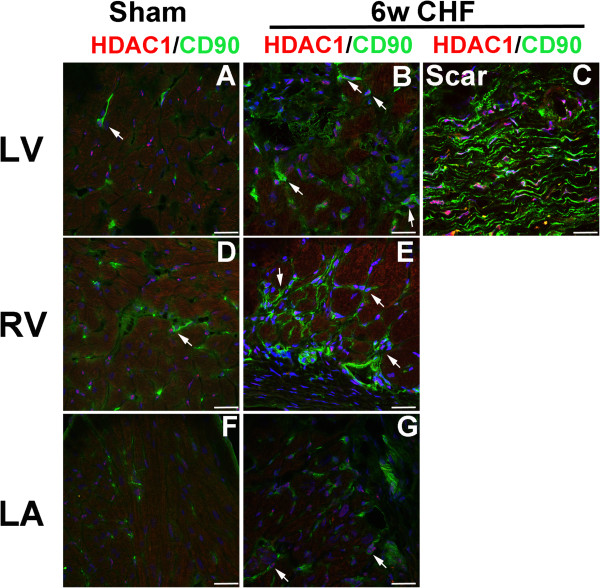
**HDAC1 is co-localized with cardiac fibroblast in the infarcted and non-infarcted myocardium in CHF.** Coronal (LA) and axial (LV, RV) sections of sham and 6w CHF hearts were stained for HDAC1 **(A-G)** and fibroblast marker CD90. DAPI (Blue) is used to stain nuclei. CD90 and HDAC1 staining were co-localized in remote LV, RV, and LA and infarcted myocardium. White arrows indicate co-localization of CD90+ cells with HDAC1. Scale bars: 100 μm. CHF, congestive heart failure; LA, left atrium; HDAC, Histone Deacetylase; LV, left ventricle; RV, right ventricle.

**Figure 5:**
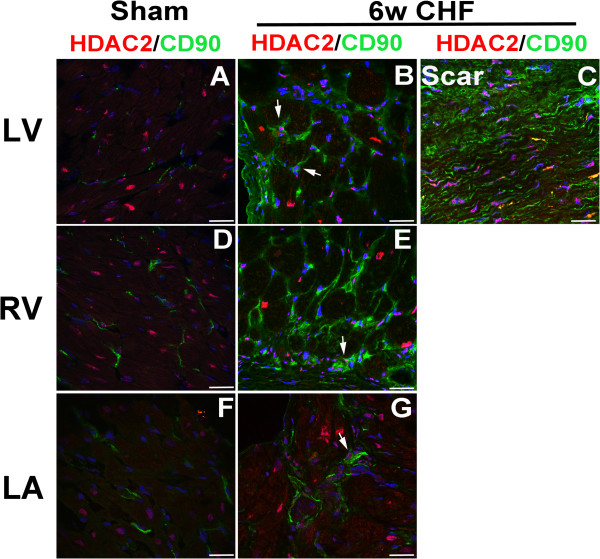
**HDAC2 is co-localized with cardiac fibroblast in the infarcted and non-infarcted myocardium in CHF.** Coronal (LA) and axial (LV, RV) sections of sham and 6w CHF hearts were stained for HDAC2 **(A-G)** and fibroblast marker CD90. DAPI (Blue) is used to stain nuclei. CD90 and HDAC2 staining were co-localized in remote LV, RV, and LA and infarcted myocardium. White arrows indicate co-localization of CD90+ cells with HDAC2. Scale bars: 100 μm. CHF, congestive heart failure; HDAC, Histone Deacetylase; LA, left atrium; LV, left ventricle; RV, right ventricle.

To investigate whether HDAC1 and HDAC2 were expressed in cardiac fibroblasts, we stained cross-sections of sham and 6w CHF hearts with fibroblast markers; CD90 and Vimentin together with HDAC1 and HDAC2. In sham hearts, CD90 (Figure 4A, D, F) and Vimentin (Additional file 1) expressing cells were distributed throughout LV, RV, and LA. In CHF, both CD90 (Figure 4B, C, E, G) and Vimentin (Additional file 1) stainings were increased in the infarcted area and in LV, RV, LA, where CD90 and Vimentin + cells were detected in patches. In the infarcted myocardium, both CD90 and Vimentin staining were co-localized with areas of dense HDAC1 and HDAC2 staining (Figure 4C and 5C). In addition, CD90 + cells were double stained with HDAC1 and HDAC2 in remote LV (Figures 4B and 5B), RV (Figures 4E and 5E), and LA (Figures 4G and 5G) of CHF hearts. Vimentin staining showed a similar pattern as CD90 in LV and RV and co-localized with HDAC1 and 2 (Additional file 1). Thus, in addition to infarcted area, CD90 and Vimentin expression and HDAC1/2 expressions are co-localized in remote LV, RV, and LA where interstitial and atrial fibrosis are observed.

**Figure 6:**
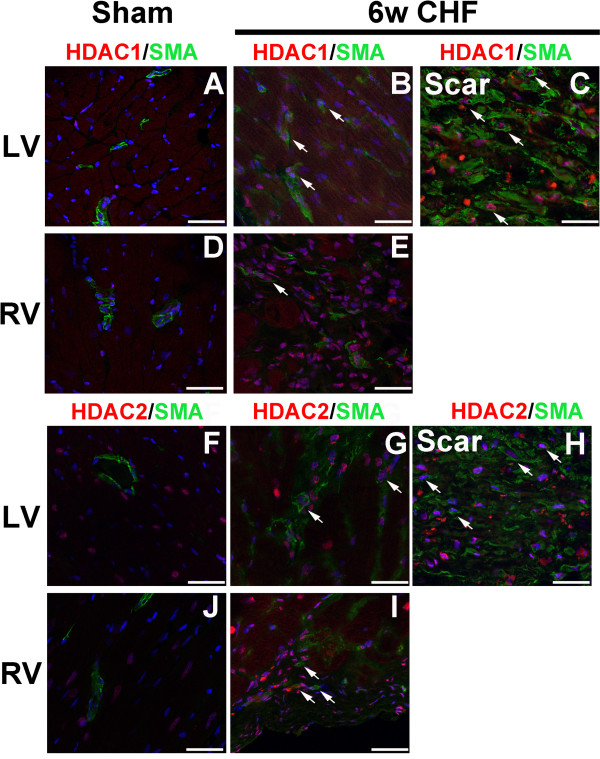
**HDAC1 and 2 are co-localized with myofibroblasts in the infarcted and non-infarcted myocardium in CHF.** Cross-sections of sham and 6w CHF hearts were stained for HDAC1 **(A-E)** or HDAC2 **(F-J)** and α-SMA. DAPI (Blue) is used to stain nuclei. α-SMA was expressed in vessels in sham LV and RV and did not co-localize with HDAC1 **(A, E)** and HDAC2 **(F-J)** staining. HDAC1 staining was co- localized with α-SMA + cells in LV **(B)** and RV **(E)** and scar **(C)** in CHF. White arrows indicate co-localized cells. Similarly, HDAC2 was co-localized with α-SMA + cells in LV **(G)** and RV **(J)** and scar **(H)** in CHF. Scale bars: 150 μm. CHF, congestive heart failure; HDAC, Histone Deacetylase; CHF, congestive heart failure; SMA, α-Smooth muscle actin.

In addition, α-SMA staining was performed to investigate whether HDAC1 and 2 staining were co-localized with myofibroblasts in 6w CHF hearts (Figure 6). While α-SMA expression is mainly observed in endothelial cells in the vessels of sham hearts, α-SMA + activated fibroblasts (myofibroblasts) emerges in MI and CHF hearts [1]. In sham hearts, α-SMA + cells were localized in blood vessels (Figure 6A, D, F, and J). In the infarcted myocardium, high level of α-SMA expression was detected in myofibroblasts and co-localized with HDAC1 and HDAC2 staining (Figure 6C and H, arrows). In addition, HDAC1/2+ α-SMA + myofibroblasts (Figure 6B, E, G, and I, arrows) were detected in the non-infarcted myocardium (LV and RV). Thus, myofibroblasts in the infarcted and non-infarcted myocardium co-express HDAC1 and HDAC2 in CHF.

### HDAC inhibition reversed myofibroblast activation *in vitro*

Activation of fibroblasts to myofibroblasts is a hallmark of cardiac fibrosis [15]. The upregulation of HDAC1 and HDAC2 primarily in cardiac fibroblasts in CHF suggests a role for HDAC1 and 2 in this process. Thus, we investigated whether HDAC inhibition reduces activation of myofibroblasts *in vitro*. We isolated CD90*+/*cKit- cardiac fibroblasts from ventricles and atria. CD90 is a surface protein expressed in cardiac fibroblasts *in vivo* and *in vitro* regardless of their activation to myofibroblasts [16]. Flow cytometry analysis confirmed that 91% of cultured CD90+ cells co-expressed α-SMA suggesting their activation to myofibroblasts in culture. In addition, they expressed myofibroblast markers; smooth muscle embryonic myosin (SMemb) and fibronectin-EIIIA variant (Additional file 2) confirming their myofibroblast phenotype. Therefore, we concluded that cultured CD90+ cells represent a suitable model to study effects of HDAC inhibition on myofibroblasts *in vitro*.

**Figure 7:**
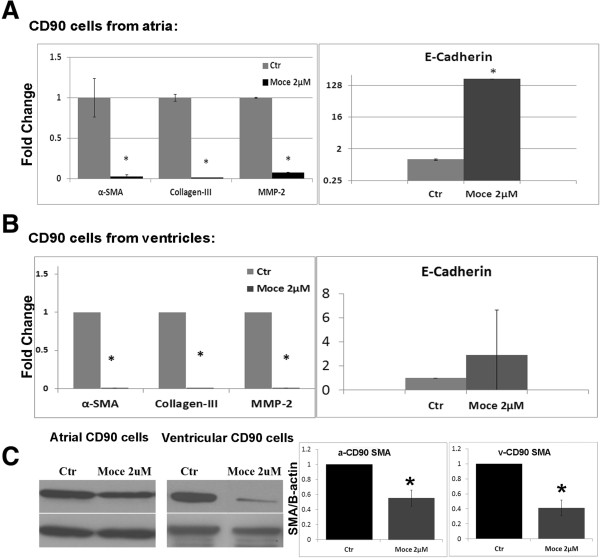
**HDAC inhibition diminished α-SMA activation in cardiac fibroblast.** CD90+ cells were isolated from atrial explants after 21 days in culture. **(A)** Cells were treated with Mocetinostat for 7 days. Relative gene expressions were measured with real-time PCR. Mocetinostat treatment elevated E-cadherin levels, while reducing α-SMA, MMP2, and Collagen-III. **(B)** CD90 cells isolated from ventricles were treated with Mocetinostat for 7 days. Levels of α-SMA, MMP2, and Collagen-III were reduced with Mocetinostat treatment, while E-cadherin expression was unaltered. **(C)** Western blot analysis of α-SMA in Mocetinostat treated atrial and ventricular CD90+ cells. Error bars indicate S.E. (n = 4). *P* <0.05. MMP2, Matrix metalloproteinase-2; Moce, Mocetinostat; SMA, α-Smooth muscle actin.

To investigate effects of class I HDAC inhibition on cardiac fibroblasts, we treated atrial and ventricular CD90+ cells with Mocetinostat, a benzamide class I HDAC inhibitor (HDAC1 (IC_50_ = 0.15 μM), HDAC2 (IC_50_ = 0.29 μM), and HDAC3 (IC_50_ = 1.66 μM) for 7 days in culture. In atrial CD90+ cells, Mocetinostat treatment resulted in downregulation of α-SMA and upregulation of E-cadherin gene expression. Gene expressions of Collagen III and MMP2, which are markers for fibrosis, were also downregulated with Mocetinostat treatment (Figure 7A).Similar to atrial CD90+ cells, Mocetinostat treatment of ventricular CD90+ cells resulted in downregulation of α-SMA, MMP2, and Collagen III gene expressions. In contrast to atrial CD90+ cells, level of E-cadherin experssion did not change with Mocetinostat treatment (Figure 7B).To confirm gene expression data, we performed western blot analysis for α-SMA (Figure 7C) in ventricular and atrial CD90+ cells. Downregulation of α-SMA protein level was observed in CD90+ cells. In conclusion, Mocetinostat treatment in atrial and ventricular fibroblasts promoted the reversal of myofibroblast phenotype to fibroblasts.

### HDAC inhibition induced E-cadherin/β-catenin expression in atrial fibroblasts

**Figure 8:**
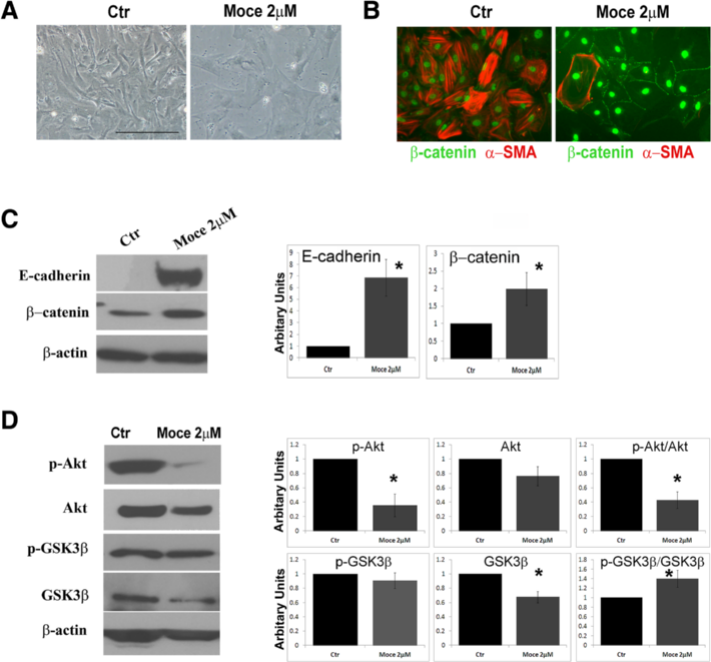
**HDAC inhibition induced MET-like process in cardiac fibroblasts.** Atrial CD90+ cells were treated with Mocetinostat for 7 days. **(A)** Transmitted light images demonstrate changes in cell morphology upon treatment with Mocetinostat. Scale bar: 100 μm. **(B)** Cells were labeled with antibodies to α-SMA (red) and β-catenin (green). DAPI (Blue) is used to stain nuclei. Scale bar: 50 μM. **(C)** CD90+ cells treated with Mocetinostat were lysed and western blot analysis was performed against E-cadherin and β-catenin. Graphs show the density analysis to corresponding to specific bands normalized to β-actin. **(D)** Western blot analysis for Akt and GSK3β signaling. Error bars indicate S.E. (n = 4) *P* < 0.05. Moce, Mocetinostat.

In atrial fibroblasts, treatment with Mocetinostat induced morphological changes (Figure 8A). To further investigate, we performed western blot against E-cadherin and β-catenin. β-catenin is a plasma membrane assiaocated protein which plays a dual role in cellular signaling. As a part of cadherin complex translocates to membrane and stabilizes cell-cell contact. As β-catenin forms a complex with E-cadherin in the plasma membrane, E-cadherin avoids its translocation to the nucleus [17]. On the other hand, when translocated to the nucleus, β-catenin promotes transcription of TCF-/LEF dependent genes. Both levels of E-cadherin and β-catenin were elevated in CD90+ cells upon Mocetinostat treatment (Figure 8C). To investigate cellular localization of β-catenin, we stained Mocetinostat treated and control CD90+ cells with β-catenin antibody (Figure 8B). In line with E-cadherin upregulation, β-catenin staining was translocated to the cell membrane upon Mocetinostat treatment. Thus, upregulated E-cadherin along with localization of β-catenin to plasma membrane suggests an initiation of a mesencymal to epithelial transformation (MET)-like process in atrial cardiac fibroblasts.

### HDAC inhibition downregulates Akt signaling

To elucidate cell signaling components associated with HDAC inhibition in cardiac fibroblasts, we measured levels of GSK3β and Akt proteins and their phosphorylation status in atrial fibroblasts *in vitro*. Akt is an important regulator of mesenchymal cell differentiation into smooth muscle cells [18] and in addition have key roles in promotion of cell proliferation, survival, and motility [19,20]. On the other hand, GSK3β is a regulator of β-catenin, where GSK3β (in unphosphorylated state) results in cytoplasmic β-catenin stabilization allowing its nuclear translocation [21].Mocetinostat treatment inactivated Akt as indicated by downregulation of p-Akt levels (Figure 8D). The level of phosphorylated GSK3β (p-GSK3β) was unchanged with Mocetinostat treatment (Figure 8D). In contrast, the total level of GSK3β was reduced. Overall, the ratio of p-GSK3β to GSK3β was elevated with Mocetinostat treatment indicating less activation of GSK3β. Thus, reduction of GSK3 β levels could be implicated in upregulation of β-catenin levels with HDAC inhibition. Thus, effects of HDAC inhibition on cardiac fibroblast could be via de-activation of Akt/GSK3 β signaling.

### Mocetinostat treatment elevates levels of p21/p53 and cleaved-caspase-3 in cardiac fibroblasts

**Figure 9:**
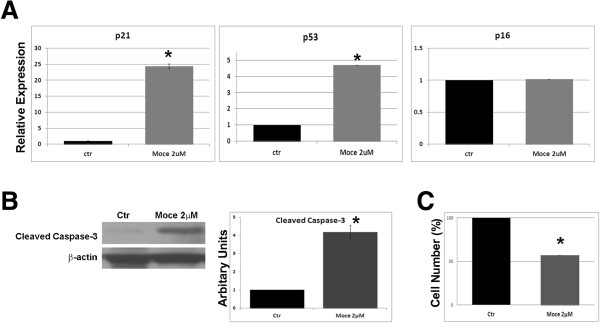
**Mocetinostat treatment elevated levels of p21/p53 and Caspase-3 in cardiac fibroblasts.** CD90+ cells were treated with Mocetinostat for 7 days. **(A)** Gene expression of p21, p53, and p16 was analyzed with real-time PCR. Mocetinostat induced expression of p21 and p53 in CD90+ cells. **(B)** Cleaved Caspase-3 levels were measured with western blot analysis. Mocetinostat induced upregulation of Caspase-3 levels in CD90+ cells. **(C)** Reduction in cell numbers upon Mocetinostat treatment was quantified by cell counting. Error bars indicate S.E. (n = 4) *P* <0.05, Moce, Mocetinostat.

We measured expression levels of p21, p53, and p16 genes which are involved in cell cycle arrest and apoptotic response. We observed upregulation of p21 and p53 genes upon treatment with Mocetinostat (Figure 9A) in atrial cardiac fibroblasts. In addition, Mocetinostat treatment caused significant reduction in cell number compared to controls (Figure 9C). In addition, we monitored the cleaved caspase 3 levels, which are associated with induction of apoptosis, by western blot (Figure 9B). At the concentration of 2 μM, Mocetinostat treatment elevated protein level of caspase-3. Thus, elevation of p21/p53 gene expression and induction of caspase-3 via Mocetinostat suggest cell cycle arrest and/or apoptosis in atrial CD90+ fibroblasts.

### Mocetinostat treatment improved cardiac function and reduced fibrosis in CHF *in vivo*

**Figure 10:**
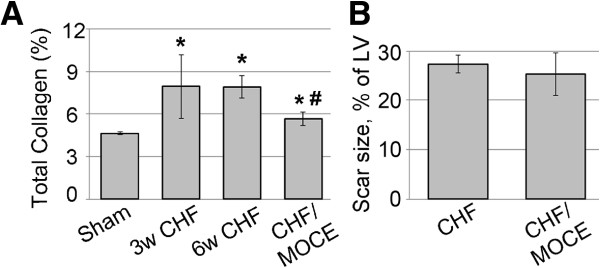
**Mocetinostat reduced fibrosis in CHF.** Total collagen percentage in sham, CHF and Mocetinostat treated CHF hearts **(A)**. Scar size analysis in Mocetinostat treated CHF hearts compared to vehicle-treated CHFs **(B)**. *, *P* <0.05 sham *vs.* CHF, sham *vs.* CHF/Moce; #, *P* <0.05, CHF *vs.* MOCE-preconditioned CHF. Moce, Mocetinostat; n = 6.

Since we observed upregulation of HDAC1 and 2 in fibroblasts and myofibroblasts in CHF infarcted area and interstitial myocardium, we tested whether selective inhibition of class I HDACs with a Mocetinostat would reduce fibrosis and improve cardiac function. We treated 3w CHF animals with 10 mg/kg of Mocetinostat daily for 3 weeks. We measured cardiac function in 6w CHF animals using a Millar conductance system (Table 1). LV end diastolic pressure and dp/dt max of Mocetinostat treated CHF rats were improved compared to CHF animals which received vehicle only. In addition, we measure total collagen amounts to assess levels of fibrosis. In 3w and 6w CHF, total collagen amount was increased compared to sham (Figure 10A). Mocetinostat treatment reduced collagen levels compared to untreated CHF group (Figure 10A). In contrast, there were no significant changes in the scar size (Figure 10B). Thus, inhibition of class I HDACs with Mocetinostat reduced collagen deposition and improved LV end diastolic pressure and dP/dtmax which are indicators of left ventricular contractility in heart failure animals.

**Table 1:** Mocetinostat improved left ventricular function in CHF

	**Heart rate (bpm)**	**Maximum pressure (mmHg)**	**End-diastolic pressure (mmHg)**	**Ejection fraction (%)**	**Cardiac output (μL/min)**	**LV dP/dt max (mmHg/s)**
CHF + MOCE	243.7 ± 21.1	110.5 ± 12.1	9.4 ± 3.4^a^	42.1 ± 14.3	23,234 ± 12,996	5,573 ± 689^a^
CHF	235.8 ± 16.1	102.7 ± 12.3^b^	28.7 ± 6.2^b^	33.6 ± 8.1^b^	19,001 ± 6,082^b^	3,921 ± 816^b^
SHAM	261.1 ± 39.4	122.5 ± 13.8	7.2 ± 2.3	63.0 ± 10.2	40,162 ± 16,010	6,928 ± 1,090

Hemodynamic parameters were measured in sham, CHF and CHF treated with Mocetinostat. Sham (n = 8), CHF (n = 10), CHF + MOCE (n = 8). Data are presented as mean ± SD.

^a^CHF + MOCE vs CHF, *P* <0.01.

^b^CHF *vs.* SHAM, *P* <0.01.

CHF, congestive heart failure; LV dP/dt_max_, peak rate of left ventricular pressure rise; MOCE, Mocetinostat.

In addition, we examined whether Mocetinostat treatment promotes apoptosis in CHF myocardium using CardioTACS *in situ* apoptosis detection kit. We did not observe increased apoptosis in scar and LV of Mocetinostat-treated CHF hearts compared to untreated CHF group (Additional file 3).

## Discussion

In this study, we demonstrated class I HDAC isoforms HDAC1 and HDAC2 are upregulated in cardiac fibroblasts as the animals progressed through heart failure. Interestingly, inhibition of Class I HDACs with Mocetinostat reversed the cardiac fibrosis in CHF animals suggesting an association between cardiac fibrosis and HDAC1/2 upregulation. Investigation of anti-fibrotic effects of HDAC inhibition on cardiac fibroblasts suggested several mechanisms including reversal of myofibroblast phenotype to fibroblast and induction of cell cycle arrest/apoptosis.

Analysis of HDAC1 and HDAC2 expression patterns in sham hearts showed that HDAC1 is mainly expressed by fibroblasts/interstitial cells while HDAC2 is more prominent in cardiomyocytes with low levels of expression in fibroblasts/interstitial cells (Figure 3). In addition, in CHF, both HDAC1 and HDAC2 showed strong staining in fibroblasts while HDAC2 maintained its expression in cardiomyocytes. Although our conclusions were based on confocal microscopy analysis, we cannot exclude the possibility of low level of expression of HDAC1 in cardiomyocytes. Altogether, these data suggest that in addition to their roles in cardiomyocyte regulation and hypertrophy [22], HDAC1 and 2 are associated with regulation of cardiac fibroblast biology in heart.

We showed that class I HDAC inhibitor, Mocetinostat improved cardiac function in parameters of ventricular contractility and reduced total collagen amount in heart failure animals. Recent studies showed that HDAC inhibition retards ventricular and atrial fibrosis formation when applied immediately after injury [23-27]. Moreover, in most of these studies pan-HDAC inhibitors such as TSA (Class I and Class II HDAC inhibitor) have been administrated for systematic HDAC inhibition. Our results highlight two important findings. First, we showed that selective inhibition of class I HDACs alone is effective in reducing fibrosis in CHF. Second, we started the treatments at 3w after MI, where animals already developed cardiac fibrosis. Thus, HDAC inhibition could effectively reverse interstitial fibrosis in heart failure and improve cardiac function.

Through HDAC inhibition with Mocetinostat *in vitro*, we observed a reduction in α-SMA, a myofibroblast marker. We showed that Mocetinostat treatment downregulated collagen III in CD90+ cells, which is consistent with previous work where TSA blocked TGFβ-induced collagen synthesis in rat cardiac fibroblasts [25]. Thus, our results suggest that the anti-fibrotic effect of Class I HDAC inhibition involves a reduction in myofibroblast activation and downregulation of one the most abundant ECM proteins, Collagen-III [28].

As existing fibroblasts differentiate into myofibroblasts, epithelial, endothelial, and smooth muscle cells can also contribute to fibrosis via EMT, EndoMT, and mechanical tension. Recent study showed that HDAC inhibition blocked TGFβ-induced EMT via upregulation of E-cadherin and downregulation of α-SMA and ECM proteins in renal injury [7]. Consistently, we showed that Mocetinostat application induced E-cadherin expression and surface translocation of β-catenin associated with morphological transformation of atrial CD90+ cells toward epithelial-like phenotype. The decrease in cell density in Mocetinostat-treated samples could induce changes in morphology of the cells as well. However, E-cadherin upregulation in these cells suggests that changes cell morphology does not solely depend on cell density. In addition, we demonstrated that in CD90+ fibroblasts, Mocetinostat reduced phosphorylation of Akt, an important EMT/EndoMT player [29-33]. Thus, another possible anti-fibrotic effect of Class I HDAC inhibition could be reversal of EMT-induced fibrosis in cardiac fibroblasts.

Other effects of HDAC inhibitors are their ability to induce cell cycle arrest and apoptosis [34]. Here, we showed a reduction in cell number, elevation of p21and p53 gene expression, and upregulation of Cleaved Caspase-3 protein with Mocetinostat treatment. p21 is a cyclin-dependent kinase inhibitor, which is involved in cell growth arrest [35], while p53 induces apoptosis. Our data are supported by the recent studies where HDAC inhibition reduced cardiac fibroblast proliferation in isoproterenol induced heart failure [24] and induced cell cycle arrest in cardiac fibroblast in model of angiotensin-II mediated fibrosis [27]. On the other hand, we did not observe an increase in overall apoptosis in CHF myocardium with Mocetinostat treatment suggesting that systemic Mocetinostat administration does not induce excessive cell death in myocardium. However, rates of apoptosis in different cells types needs to be examined to determine whether Mocetinostat induces apoptosis selectively in certain cell types, that is, fibroblasts *in vivo.* Altogether, these data suggest that regulation of fibroblast proliferation and apoptosis could be another possible mechanism of HDAC inhibition in control of fibrosis.

One of the limitations in our study is that we isolated cardiac fibroblast from atrial explants and ventricular tissue based on CD90 (Thy1) surface marker. Even though the majority of cardiac fibroblasts express CD90, this marker is not fibroblast-exclusive. Thus, other cells types such as blood originated cells could be included as well. However, a fibroblast specific marker representing majority of the fibroblasts in the heart is not currently available. In addition, CD90+ cells do not represent entire fibroblast population in heart; this marker is rather expressed on a subset of fibroblasts. Therefore, we used another marker, Vimentin (also not fibroblast specific), to confirm expression of HDAC1 and HDAC2 in cardiac fibroblasts. Thus, expression of HDAC1/2 in both CD90 and Vimentin + cells suggests that cardiac fibroblasts express HDAC1/2 in CHF hearts.

## Conclusions

Our results show that cardiac fibroblasts activated HDAC1 and 2 in infarcted/non-infarcted myocardium and atrium in CHF. In addition, inhibition of Class I HDACs in cardiac fibroblasts reduces myofibroblasts activation*.* This knowledge is important to further understand more fibroblast-specific biology HDAC inhibition and develop specific therapeutic agents to reduce fibrosis.

## Methods

### Animals

This study was performed in an accredited facility by the American Association for Accreditation of Laboratory Animal Care and was approved by the Institutional Animal Care and Use Committee at Banner Sun Health Research Institute. Animals received humane care in compliance with the *Guide for the Care and Use of Laboratory Animals* published by the US National Institutes of Health (NIH Publication No. 85–23, revised 1996).

### Myocardial infarction and treatments

MI was created by ligation of the left coronary artery as previously performed by our laboratory [36]. In brief, rats were anesthetized using 1 mL/kg of an MI cocktail composed of ketamine (50 mg/mL), xylazine (15 mg/mL), acepromazine (2 mg/mL), and atropine (1 mg/mL). Animals were intubated and ventilated using a small animal ventilator (Harvard Apparatus). A left thoracotomy was performed via the third intercostal rib, and the left coronary artery was ligated. In sham operated animals, the chest was closed without ligation of the artery. The rats were sacrificed at 3 days (Acute MI-AMI), 3 weeks, or 6 weeks (congestive heart failure (CHF)) post surgery. At 3 weeks or 6 weeks after successful infarction, rats exhibited CHF indicated by elevation of left ventricular end-diastolic pressure (LVEDP), LV remodeling, and fluid accumulation in the chest [36,37]. In addition, prominent fibrosis in the ventricles and left atrium was evident at 6 weeks post surgery. Closed-chest *in-vivo* cardiac function was measured using a Millar pressure conductance catheter system (Millar instruments, Houston, TX, USA) as previously described [38]. A transverse cut was made to postmortem hearts to assess scar size and LV remodeling. Only animals with visible scar, LV remodeling, and LVEDP greater than 20 mmHg were included in the 3-week or 6-week CHF group.

Three weeks post surgery, animals in the CHF group were further divided into two groups; the first group received 10 mg/kg Mocetinostat dissolved in 0.1 N HCl PBS solution daily for the duration of 3 weeks (n = 8). The second group received only vehicle for the same duration (n = 6). In addition, animals that underwent sham surgery were injected with vehicle only and served as control group (n = 5).

### Cell isolation and culture

Atrial explant outgrowth was generated as previously described [38,39]. Briefly, tissue was cut into 1 to 2 mm^3^ pieces and digested with 0.2% trypsin (Life Technologies, Carlsbad, CA, USA) and 0.1% collagenase IV (Life Technologies) for a total of 10 min. The remaining tissue fragments were cultured as ‘explants’ in explants medium (CEM), which was composed of IMDM supplemented with 10% fetal bovine serum (FBS, Lonza), 100 U/mL penicillin G, 100 μg/mL streptomycin, and 2 mmol/L L-glutamine (Sigma-Aldrich). After 21 days in culture, cells were collected by trypsinization. CD90+ cells were separated from the cell outgrowths using magnetic beads (MACS, Miltenyi Biotec) according to manufacturer protocol and analyzed by flow cytometry for purity assessment. CD90 cells from ventricles were isolated by enzymatic digestion with a Dispase II (2.4 mg/mL, Roche)/Collagenase II (0.05 mg/mL, Gibco) mix in PBS. Ventricles were cut into small pieces and digested for 10 min at 37°C with agitation. The supernatant spun at 1,200 g for 7 min to collect dissociated cells. The last two steps were repeated five times and cells were pooled. Dissociated cells were plated and media was changed after 2 h to discard non-attached cells and debris. Then, attached cells were trypsinized and collected for CD90 isolation as described above.

Isolated atrial or ventricular CD90+ cells were seeded on six well plates at a density of 0.2 × 10^6^ cells/well in CEM. Cells were treated with 1 μM and 2 μM of Mocetinostat (SelleckChem). Control cells were treated with 0.005% DMSO.

### Immunostaining

Heart tissue was embedded in tissue freezing media (Triangle Biomedical Science) snap-frozen in liquid nitrogen and sectioned in the coronal plane using Leica CM1900 cryostat (Leica Microsystems, Bannockburn, IL, USA). Coronal and axial tissue sections (5 to 7 μm thickness) were mounted on positively charged glass slides and fixed/permeabilized in a 1:1 mixture of acetone and ethanol. Sections were blocked with 3% BSA in PBS and incubated with primary antibodies against α-MHC (Abcam), HDAC1, HDAC2, α-SMA (Abcam), CD90 (BD), and Vimentin (Abcam). Specific staining was visualized using corresponding secondary antibodies conjugated with Alexa 488 or 568 (Molecular Probes). Nuclei were stained with DAPI, 4′ 6-diamidino-2-phenylindole (Life Technologies).

Cells were fixed/permeabilized in a 1:1 mixture of acetone and ethanol. Cells were blocked with 3% BSA in PBS and stained with primary antibodies. Corresponding secondary antibodies were conjugated with Alexa-488 or Alexa 568 (Molecular Probes). Nuclei were stained with DAPI, 4′ 6-diamidino-2-phenylindole (Life Technologies).

Fluorescent images were captured using Leica TCS SPE confocal system configured with Leica DM 2500 microscope. Excitation maximums of 488 nm, 532 nm, and 405 nm, were used for image acquisition. Images were processed using LAS AF software (Leica Microsystems).

### Scar size assessment and collagen assay

Heart tissue was processed as described above and sections of 7 or 20 μm were mounted on positively charged glass slides. Routine staining was performed with the Hematoxylin and Eosin kit (H&E, Sigma) according to manufacturer instructions. For scar size assessment, sections were stained with Masson’s Trichrome kit (Sigma-Aldrich) according to the manufacturer’s protocol. Transmitted light images of heart sections were processed using DP2-BSW software (Olympus Corp). Scar percentage was calculated as a ratio of collagen enriched scar area (blue staining) to the whole left ventricle area (red staining).

Total collagen amount was measured in 20 μm cross-sections using Sirus Red Fast Green Collagen staining kit (Chondrex) according to manufacturer instructions. Absorbance of collagen (540 nm) and non-collagenous protein (605 nm) was assessed using BioTek Synergy HT Microplate Reader. Collagen percentage was calculated as a ratio of OD540_collagen_ to OD605_non-collagenous protein_.

### RNA isolation and quantitative real-time RT-PCR

Total RNA was extracted from CD90+ cells using PureLink™ RNA Mini Kit (Life Technologies) according to the manufacturer’s protocol. RNA was then quantified with the Quanti-iT™ RiboGreen® RNA Assay Kit, and assessed using BioTek Synergy HT Microplate Reader (excitation/emission 480 nm/520 nm). Total RNA (200 ng) was reverse transcribed with QuantiTect Reverse Transcription kit (Qiagen). Real-time RT-PCR was conducted using the Rower SYBR Green Master Mix (Applied Biosystems) on a StepOnePlus Real-time PCR System (Applied Biosystems). Specific primers were synthesized by Life Technologies (sequences are available upon request). CYP A was used as a reference gene. Data analysis was performed on StepOne software version 2.1 (Applied Biosystems) using the comparative Ct (ΔΔCt) quantitation method.

### Western blotting

Scar and LV tissue were dissected from AMI, CHF, and sham hearts, respectively. Dissected tissues and isolated cells were homogenized in lysis buffer (50 mM Tris- HCl pH7.5, 150 mM NaCl, 0.5% NP-40 (Sigma), 0.5% Triton-X (Sigma), 1 mM EDTA (Sigma), and complete mini protease inhibitor (Roche). Protein concentrations were determined by BCA assay (Thermo Scientific). Typically, 40 μg of protein was loaded on 4% to 12% Tris-Bis gels (Life Technologies), separated in MOPS running buffer, and transferred to a PVDF membrane (Millipore). After blocking with 5% non-fat dry milk in 1× TBS, membranes were probed with HDAC1, HDAC2 (Abcam, 1:1,000), α-SMA (Sigma, 1:5,000), E-cadherin, GSK3β (Santa Cruz, 1:500), p-GSK3β, Cleaved Caspase 3, p-Akt, Akt (Cell Signaling, 1:1,000), β-Catenin active (Millipore,1:1,000), and β-Actin (Sigma, 1:50,000) in TBS with 5% milk overnight at 4°C. Following three washes in TBS, membranes were incubated with HRP-conjugated secondary antibodies (Santa Cruz, 1:50,000) for 1 h at room temperature. An ECL (Millipore) system was used for detection of the bands and exposed to X-ray film (Thermo Scientific) in a dark room. Densitometry analysis was performed with Alpha Ease FC software.

### Statistical analysis

All datasets are represented as mean ± S.E. Significance (*P* <0.05) was determined using Student’s *t*-test. Statistical analysis was conducted using Sigma Stat 3.5 software.

## Abbreviations

CHF: Congestive heart failure; EMT/EndoMT: Epithelial/endothelial mesenchymal transition; HDAC: Histone deacetylase; LA: Left atrium; LV: Left ventricle; MI: Myocardial infarction; MMP2: Matrix metalloproteinase-2; MOCE: Mocetinostat; RV: Right ventricle; SMA: α-smooth muscle actin; TSA: Trichostatin A; α-MHC: Myosin heavy chain.

## Competing interests

The authors declare that they have no competing interests.

## Authors’ contributions

HNG conceived and designed the experiments, performed western blotting, immunohistochemistry, and data analysis, and wrote the manuscript. LZ performed immunohistochemistry and contributed to writing of the manuscript. JN performed animal surgeries and cardiac function analysis. SP performed and participated in *in vitro* experiments. DM helped in design of the study. MG conceived and designed the experiments and helped in writing of the manuscript. All authors read and approved the final manuscript.

## Supplementary Material

Additional file 1 **HDAC1 and 2 are co-localized with cardiac fibroblast in the infarcted and non-infarcted myocardium in CHF.** Coronal (LA) and axial (LV, RV) sections of sham and 6w CHF hearts were stained for HDAC1 (A-E) or HDAC2 (F-I) and Vimentin. Scale bars: 150 μm. CHF, congestive heart failure; HDAC, Histone Deacetylase; LV, left ventricle; RV, right ventricle. Click here for file

Additional file 2 **CD90+ cells express myofibroblast markers.** (A) Flow cytometry analysis of CD90+ cells. CD90+ cells were fixed in 70% ethanol and double labeled with anti-CD90 antibody conjugated with FITC (BD Biosciences) and mouse anti-Vimentin or mouse anti-SMA antibodies following by labeling with anti-mouse IgG conjugated with PE-Cy5.5 (Life Technologies). For a negative control, cells were labeled with isotype IgG instead of primary antibody. Cell events were detected using FACS Calibur flow cytometer equipped with argon laser (BD Biosciences). Data were analyzed using CellQuest software (BD Biosciences). (B) CD90 cells isolated from both ventricles and atria express SMemb and Fn-EIIIA under culture conditions described in material and methods section. Fn-EIIIA, Fibronectin-EIIIA variant; SMemb, Smooth muscle embryonic myosin. Click here for file

Additional file 3 Mocetinostat treatment does not elevate apoptosis in CHF myocardium. Apoptotic cells were stained with CardioTACS *in situ* apoptosis detection kit (Trevigen) following manufacturer’s instructions in both Mocetinostat treated and untreated CHF tissue sections. Briefly, tissue sections were fixed with 4% formaldehyde. Apoptosis assay was performed *in situ* by incorporating labeled nucleotides onto free 3′ OH ends of DNA fragments using a terminal deoxynucleotide transferase enzyme. Streptavidin-horseradish peroxidase was used to detect biotinylated nucleotides incorporated. A dark blue precipitate was generated by reaction with TACS Blue label and visualized under light microscope. Arrows indicate positive cells for apoptosis. Click here for file
