# Supplementary figures and images for: HDAC class I inhibitor, Mocetinostat, reverses cardiac fibrosis in heart failure and diminishes CD90+ cardiac myofibroblast activation
Source: Fibrogenesis Tissue Repair. 2014 Jul 2;7:10. doi: 10.1186/1755-1536-7-10 (PMC4094898; doi:10.1186/1755-1536-7-10)

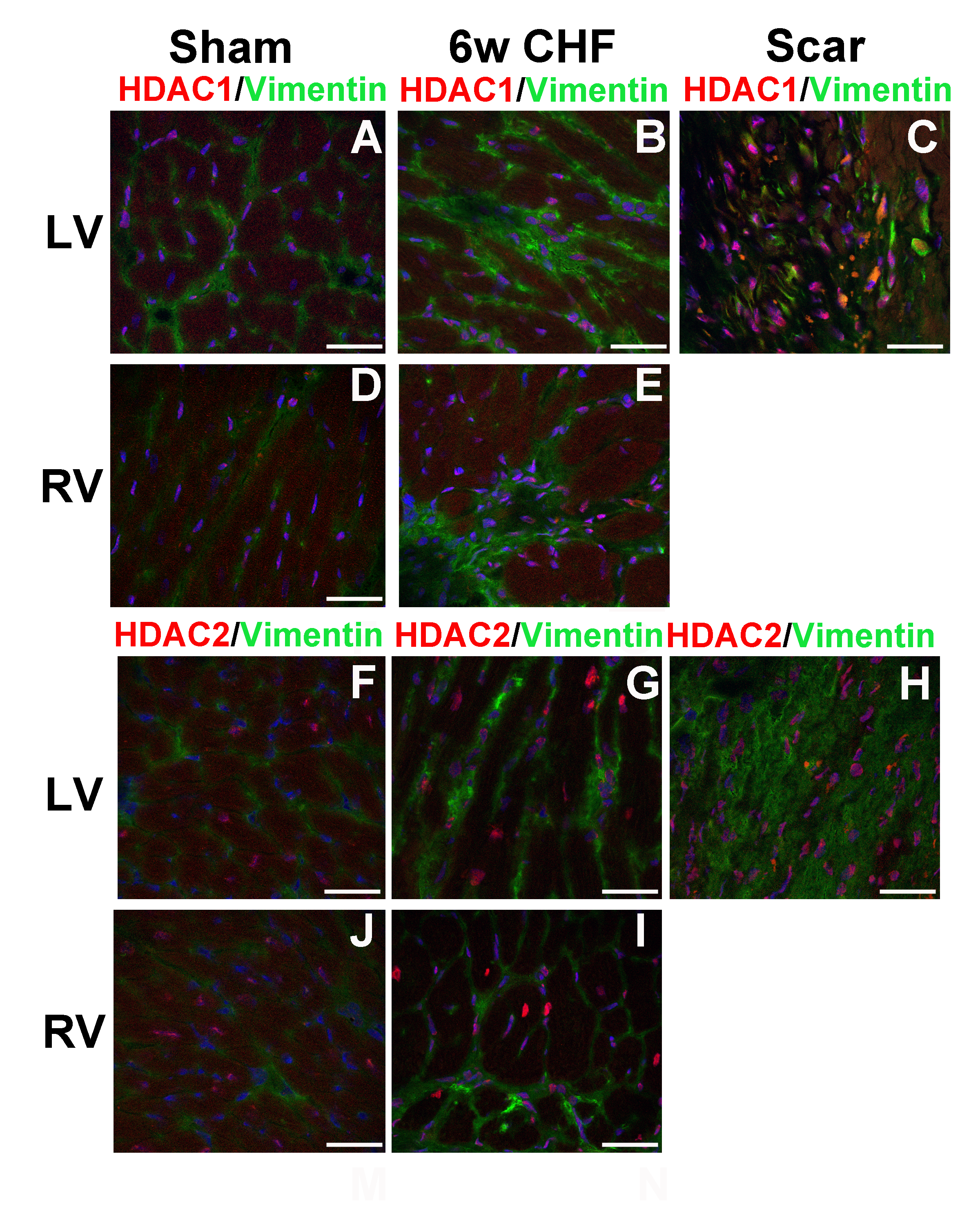

Supplement: Additional file 1 — HDAC1 and 2 are co-localized with cardiac fibroblast in the infarcted and non-infarcted myocardium in CHF. Coronal (LA) and axial (LV, RV) sections of sham and 6w CHF hearts were stained for HDAC1 (A-E) or HDAC2 (F-I) and Vimentin. Scale bars: 150 μm. CHF, congestive heart failure; HDAC, Histone Deacetylase; LV, left ventricle; RV, right ventricle. [file 1755-1536-7-10-S1.tiff]

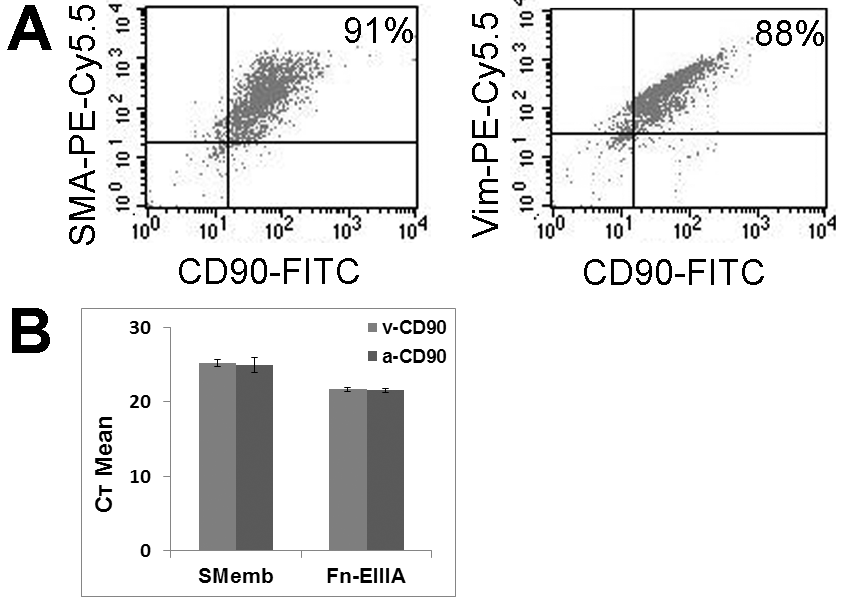

Supplement: Additional file 2 — CD90+ cells express myofibroblast markers. (A) Flow cytometry analysis of CD90+ cells. CD90+ cells were fixed in 70% ethanol and double labeled with anti-CD90 antibody conjugated with FITC (BD Biosciences) and mouse anti-Vimentin or mouse anti-SMA antibodies following by labeling with anti-mouse IgG conjugated with PE-Cy5.5 (Life Technologies). For a negative control, cells were labeled with isotype IgG instead of primary antibody. Cell events were detected using FACS Calibur flow cytometer equipped with argon laser (BD Biosciences). Data were analyzed using CellQuest software (BD Biosciences). (B) CD90 cells isolated from both ventricles and atria express SMemb and Fn-EIIIA under culture conditions described in material and methods section. Fn-EIIIA, Fibronectin-EIIIA variant; SMemb, Smooth muscle embryonic myosin. [file 1755-1536-7-10-S2.tiff]

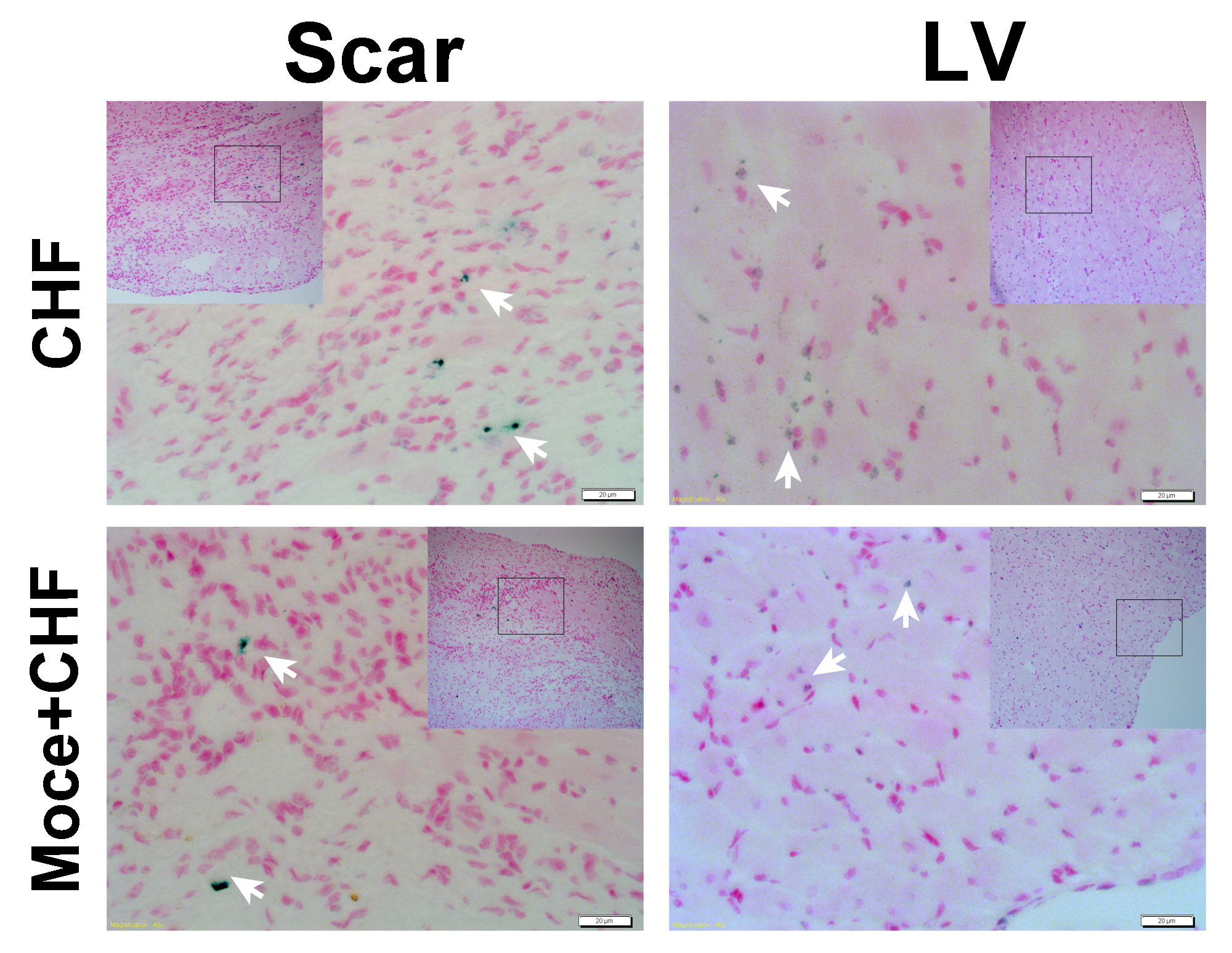

Supplement: Additional file 3 — Mocetinostat treatment does not elevate apoptosis in CHF myocardium. Apoptotic cells were stained with CardioTACS in situ apoptosis detection kit (Trevigen) following manufacturer’s instructions in both Mocetinostat treated and untreated CHF tissue sections. Briefly, tissue sections were fixed with 4% formaldehyde. Apoptosis assay was performed in situ by incorporating labeled nucleotides onto free 3′ OH ends of DNA fragments using a terminal deoxynucleotide transferase enzyme. Streptavidin-horseradish peroxidase was used to detect biotinylated nucleotides incorporated. A dark blue precipitate was generated by reaction with TACS Blue label and visualized under light microscope. Arrows indicate positive cells for apoptosis. [file 1755-1536-7-10-S3.tiff]
